# The role of psychological distress in the relationship between lifestyle and compulsivity: An analysis of independent, bi-national samples

**DOI:** 10.1017/S1092852921001048

**Published:** 2021-12-13

**Authors:** Mary-Ellen E. Brierley, Lucy Albertella, Kristian Rotaru, Louise Destree, Emma M. Thompson, Chang Liu, Erynn Christensen, Amelia Lowe, Rebecca A. Segrave, Karyn E. Richardson, Edouard Kayayan, Samuel R. Chamberlain, Jon E. Grant, Rico S.C. Lee, Sam Hughes, Murat Yücel, Leonardo F. Fontenelle

**Affiliations:** 1 BrainPark, Turner Institute for Brain and Mental Health, School of Psychological Sciences & Monash Biomedical Imaging Facility, Monash University, Clayton, VIC, Australia; 2 Monash Business School, Monash University, Caulfield, VIC, Australia; 3 Department of Psychiatry, University of Southampton, Southampton, United Kingdom; and Southern Health NHS Foundation Trust, Southampton, United Kingdom; 4 Department of Psychiatry and Behavioural Neuroscience, University of Chicago, Chicago, IL, United States; 5 Obsessive, Compulsive, and Anxiety Spectrum Research Program, Institute of Psychiatry, Federal University of Rio de Janeiro (UFRJ); 6 D’Or Institute for Research and Education, Rio de Janeiro, Brazil

## Abstract

**Objective:**

Poor mental health is a state of psychological distress that is influenced by lifestyle factors such as sleep, diet and physical activity. Compulsivity is a transdiagnostic phenotype cutting across a range of mental illnesses including obsessive-compulsive disorder, substance-related and addictive disorders, and is also influenced by lifestyle. Yet, how lifestyle relates to compulsivity is presently unknown, but important to understand to gain insights into individual differences in mental health. We assessed (1) the relationships between compulsivity and diet quality, sleep quality and physical activity, and (2) whether psychological distress statistically contributes to these relationships.

**Methods:**

We collected harmonised data on compulsivity, psychological distress, and lifestyle from two independent samples (Australian n = 880, US n = 829). We used mediation analyses to investigate bi-directional relationships between compulsivity and lifestyle factors, and the role of psychological distress.

**Results:**

Higher compulsivity was significantly related to poorer diet and sleep. Psychological distress statistically mediated the relationship between poorer sleep quality and higher compulsivity, and partially statistically mediated the relationship between poorer diet and higher compulsivity.

**Conclusion:**

Lifestyle interventions in compulsivity may target psychological distress in the first instance, followed by sleep and diet quality. As psychological distress links aspects of lifestyle and compulsivity, focusing on mitigating and managing distress may offer a useful therapeutic approach to improve physical and mental health. Future research may focus on the specific sleep and diet patterns which may alter compulsivity over time to inform lifestyle targets for prevention and treatment of functionally impairing compulsive behaviours.

## Introduction

Compulsivity is the tendency to engage in repetitive, habitual or ritualistic behaviours [1]. Such behaviours are carried out by individuals to abide by existing cognitive rules and the sense that these behaviours must be completed, despite the individual’s awareness that these behaviours do not align with their overall goals [2]. High compulsivity is correlated with a number of psychiatric disorders. Conditions characterised by high compulsivity include, but are not limited to clinical and sub-clinical symptoms of obsessive-compulsive disorder (OCD), body dysmorphic disorder, hoarding disorder (HD), trichotillomania (hair pulling disorder), excoriation (skin-picking) disorder, and disorders due to addictive behaviours such as substance use and gambling disorders [3, 4]. As well as compulsivity, increased psychological distress has been shown to be a key driver of transdiagnostic compulsive behaviours in community samples [5, 6].

The poor physical health of people living with mental illness has been reported globally, regardless of socioeconomic status [7]. While this field of research has assessed an array of factors, those lifestyle factors commonly linked with mental health include diet quality, sleep quality, and physical activity [8]. Disorders involving compulsive behaviours are no exception, and the high prevalence of unhealthy lifestyle behaviours of individuals with compulsive disorders has been well documented [9].

For instance, a review of sleep in obsessive-compulsive and related disorders (OCRDs) detected a general trend of increased sleep disturbance in people experiencing OCRDs, with sleep disturbance being more common in OCD and HD [10]. Further, higher sugar and fat intake is shown to be significantly associated with greater gambling pathology [11], while robust randomised controlled trials have shown the nutrient supplement n-acetylcysteine (commonly found in protein-rich foods) to improve symptoms across OCRDs spectrum alongside standard treatment [12–15]. While many studies have relied on cross-sectional data and we therefore cannot infer causal association between lifestyle and compulsive behaviours, causal links between diet factors and OCD-like behaviours have been shown in animal models [16, 17]. Research has suggested that individuals with OCD (and without depression) present with a less varied gut micro-biome and higher inflammation levels when compared to a non-psychiatric control group [18, 19]. This evidence supports theoretical models linking the microbiome – influenced by a range of lifestyle factors - and compulsivity [17, 20].

The evidence surrounding physical activity and compulsivity remains mixed. For instance, cross-sectional studies have not revealed a relationship between exercise and OCD [21] and, in fact, alcohol use disorder (excluding severe forms) has been associated with higher levels of physical activity [22]. Yet, acute decreases in OCD symptoms have been reported by participants of moderate intensity physical exercise interventions [23, 24] (for a review see [12]). More generally, physical activity is positively associated with broad mental health outcomes including anxiety and depression [25, 26]. Further research is needed in a general population to tease apart the relationships between mental health or psychological distress, physical activity and compulsivity.

Emerging evidence indicates lifestyle factors play a key role in general mental wellbeing through common mechanisms of action which influence psychological distress in community and clinical samples. Low levels of brain-derived neurotropic factor (BDNF; an important protein for neuroplasticity and mental wellbeing) have been reported amongst individuals engaging in compulsive behaviours [27, 28]. To counter this, BDNF levels can be increased - and, potentially, compulsivity may be decreased - through engagement in high-intensity physical activity [29], diet quality [30, 31] and sleep quality improvements through a reduction in psychological distress [32, 33]. Analysing these lifestyle factors, especially in combination, will be valuable in understanding whether they impact similar mental health constructs such as psychological distress and compulsivity. As lifestyle and mental health factors can differ across cultures, studying these relationships in multiple independent samples is important in understanding whether there may be a consistency across the mechanisms influencing the relationship between lifestyle and compulsivity.

Psychological distress has been reliably linked to a number of lifestyle factors including diet quality and dietary patterns, including adherence to a Mediterranean diet [34–36], amount of physical activity [37, 38] and sleep quality [39]. Yet, the direction of the relationship between compulsivity and lifestyle requires further investigation. It is unknown, for instance, whether unhealthy/poor quality lifestyle behaviours increase compulsivity directly (through an effect on cognitive mechanisms underlying behaviour), whether compulsivity leads to unhealthy/poor quality lifestyle behaviours by interfering directly with healthier daily activities (e.g., less sleep time due to compulsions being carried out late into the evening) [9], or whether this relationship may be mediated by other factors, including psychological distress. This holds important implications for therapeutic targets for preventing and treating compulsive disorders, as well as informing the true transdiagnostic nature and potential of lifestyle-based interventions.

This study has two aims: (i) To assess the relationship between lifestyle factors (specifically diet quality, sleep quality and level of physical activity) and transdiagnostic compulsivity; and (ii) To assess whether the relationship between lifestyle factors and compulsivity is statistically accounted for by psychological distress. Considering that lifestyles, compulsivity levels and distress related to these factors may be different across cultures, we attempted to determine the reliability and generalisability of our results in a cross-sectional design using two independent community samples.

## Methods

We conducted two independent, online, cross-sectional questionnaire studies in accordance with the Declaration of Helsinki. The Monash University Human Research Ethics Committee reviewed and approved both studies.

### Participants

Across both samples, eligible participants were adults (18 years and over).

## Sample One – United States

As reported previously [40, 41], we recruited 829 participants residing in the United States of America in July 2020 through Amazon Mechanical Turk, an online crowdsourcing platform targeting US participants. Participants were reimbursed US$15. We restricted our recruitment to participants whose first language was English or who reported learning English before the age of seven, and used the Approved Participants function on CloudResearch to only include participants who have passed previous attention and engagement measures distributed by the platform.

## Sample Two - Australia

As reported previously [5], we recruited 992 participants throughout Australia from May-July 2020 primarily through Prolific (n=698) - an online crowdsourcing platform - and used the Custom Pre-screening function to recruit only participants residing in Australia. The remaining participants (n=294) were recruited through social media advertisements. Participants recruited through Prolific received reimbursement of £7.50 per hour and offered participants completing the questionnaire through other means, entry into a draw to win one of 50 AU$100 vouchers for a department store.

### Measures

Participants in both samples provided demographic information including their age, sex and employment status, and completed a series of questionnaires related to psychiatric history, personality factors, lifestyle and obsessive-compulsive symptoms (Table 1 and outlined in text below). Unless stated otherwise, the below measures were applied across both samples.

#### Compulsivity

##### Cambridge-Chicago Compulsivity Scale (CHI-T)

The CHI-T measures compulsivity across 15 statements [42]. Item response options range from strongly disagree (0) to strongly agree (3). The CHI-T total score – the score of interest for this study - ranges from 0 to 45 with higher scores indicating higher compulsivity. The CHI-T shows concurrent validity with the Padua Inventory (a scale for the measurement of Obsessive-Compulsive symptoms) and the Structured Clinical Interview for Gambling Disorder [42]. The CHI-T is often used as a measure of symptom severity across transdiagnostic compulsive disorders [43].

#### Impulsivity

##### Short Form of the Barrett Impulsivity Scale-15 (BIS-15)

The BIS-15 is a 15-item scale measuring impulsivity. It has been widely used in community samples and shown a 3-factor structure of non-planning, motor impulsivity, and attention impulsivity. Psychometric properties support the BIS-15 as a valid and reliable measure of impulsivity [44]. We used the BIS-15 total score (15-60), and this measure was completed by Sample One only.

##### Short Urgency, Premeditation, Perseverance, Sensation Seeking, Positive Urgency, Impulsive Behavior Scale (S-UPPS-P)

The S-UPPS-P is a 20 item measure with five subscales relating to different domains of impulsivity [45]. Participants are required to rate their level of agreement with statements regarding how they generally think and act. Total scores range from 20-80 where higher scores indicate greater levels of impulsivity. We referred to the UPPS-P total score, and this was completed by Sample Two only.

#### Psychological distress

##### Depression, Anxiety and Stress Scale-21 (DASS-21)

The DASS-21 is an established measure of psychological functioning [46] through negative emotional states of depression, anxiety and stress [47]. Participants select a response from 0 (‘did not apply to me at all) – 3 (applied to me very much or most of the time) response indicating how they have felt in the past week. The DASS-21 total score was the measure of interest for our study, and this measure was used in the first study only.

##### K10

The K10 is a 10-item measure of psychological distress over the past month [48]. Participants are asked to rate their level of distress (e.g., “About how often did you feel hopeless”) on a five-point Likert scale. The K10 total score was the measure of interest for our study, and this measure was used for Sample Two only.

#### Lifestyle factors

##### Pittsburgh Sleep Quality Index (PSQI)

The PSQI measures sleep quality considering several domains: subjective sleep quality, sleep latency, sleep duration, habitual sleep efficiency, sleep disturbance, use of medication and daytime dysfunction [49]. Scoring is based on a four-point Likert scale and total scores range from 0-42 with higher scores indicating poorer sleep quality. The PSQI demonstrates high internal consistency (α=0.83). We referred to the total score in the current study.

##### Short Form Food Frequency Questionnaire (SFFFQ)

The SFFFQ measures diet quality based on the respondent’s reported food and drink intake over a typical week in the past month [50]. Responses are given on a 6- to 8-point Likert scale ranging from ‘rarely or never’ to ‘5+ a day’or’7+ a week’. The SFFFQ was developed for use in a British population, however, we adapted the measure and employed language more appropriate to use in the United States of America. Data from the SFFFQ is represented by an overall Diet Quality Score (DQS) – the score of interest for this study - with a highest possible score of 15.

##### International Physical Activity Questionnaire (IPAQ)

The IPAQ is a short measure of physical activity based on participant recall from the seven days prior [51]. Participants are asked to provide the number of days they engaged in vigorous intensity physical activity, moderate intensity physical activity and walking, with example of each of these provided (e.g., “During the last 7 days, on how many days did you do vigorous physical activities like heavy lifting, digging, aerobics, or fast bicycling?”). Participants are also asked to record how long they usually spent doing each of these activities, with reference to one of the days (e.g., “How much time did you usually spend doing vigorous physical activities on one of those days?”). Participants are also asked to provide their average sitting time each day. Vigorous, moderate and walking physical activity is converted to metabolic equivalent of task (MET) minutes and totalled to provide a single MET minutes scores for each participant. This version of the IPAQ was for Sample One.

In the second study, we used an adapted version of the IPAQ wherein participants who recorded any days of vigorous, moderate or low intensity activity were asked to state the estimated number of minutes they were engaged in the respective activities for each of these days. We then converted recorded physical activity time to MET minutes.

### Data analysis

We performed statistical analyses using SPSS Version 26 and the PROCESS macro (see below for further statistical details) [52]. We obtained descriptive statistics for each sample (age, sex and employment status), and undertook analyses for the two studies independently from one another.

At the data analysis stage, we removed participants who did not complete all study measures relevant to our analyses. Following removal of cases with missing data, we independently analysed data from 749 participants in Sample One and 796 participants in Sample Two. We ran independent samples t-tests, or chi-square tests where appropriate, to test for statistically significant differences between the two groups in terms of age, gender, employment, compulsivity and lifestyle factors. Due to differing instruments used to measure impulsivity and psychological distress, it was not possible to test for a statistically significant difference between groups on these constructs.

We performed six mediation analyses on each sample. Initially, sleep quality, diet quality and physical activity formed predictor variables. Psychological distress was defined as a candidate mediating variable and compulsivity was the outcome variable. As we used crosssectional data in which causality cannot be assumed, we also ran mediation analyses with sleep quality, diet quality and physical activity forming outcome variables, compulsivity being the predictor variable and psychological distress remaining the mediator.

We controlled for impulsivity, age and sex across all analyses by adding these variables into the mediation models as covariates. Of note, we controlled for impulsivity as a possible confound on the relationships between lifestyle, psychological distress and compulsivity. Past research has shown impulsivity to be highly correlated with compulsivity and to influence psychological distress [1, 41, 53, 54]. We adopted an error rate of p<0.05 to determine significance of total and direct effects in the mediation analyses, and the bootstrapping method to determine significance of the indirect effect, and therefore the existence of mediation in the model. Using the bootstrapping method, the indirect effect is deemed significant when the confidence interval of the bootstrapped distribution does not pass through zero [55]. We referred to the unstandardised coefficient beta (*b*) to determine effect sizes. In this study, we used the CHI-T, typically a measure of compulsivity, as an indicator of transdiagnostic symptom severity. To confirm that data from both samples are consistent with previous research stating that higher compulsivity is associated with greater compulsive psychopathology [42], we ran the same mediation analyses as stated above, replacing the CHI-T with a measure of obsessive-compulsive symptom severity (that is, the DOCS from Sample One and the OCI-R from Sample Two). Please see Supplementary material for these results.

## Results

Please refer to Table 2 for participant characteristics. Compared to participants in Sample One, those in Sample Two were significantly younger in age, less likely to be employed full-time, more likely to endorse another form of employment (e.g., casual or part-time employment, or study) or to be unemployed. Additionally, more participants in Sample Two identified as a gender other than male or female than those in Sample One. Participants in Sample Two reported significantly higher levels of compulsivity, higher diet quality and less physical activity than those in Sample One.

We present results of mediation analyses below with lifestyle factors as predictors of compulsivity in Table 3, and compulsivity as a predictor of lifestyle factors in Table 4. Please also see supplementary material for mediation analyses examining the relationship between obsessive-compulsive symptom severity (DOCS and OCI-R) and lifestyle factors. Generally, examination of the relationship between obsessive-compulsive symptom severity and lifestyle factors mirrored findings from our compulsivity analyses.

### Sleep and compulsivity

**Figure 1:**
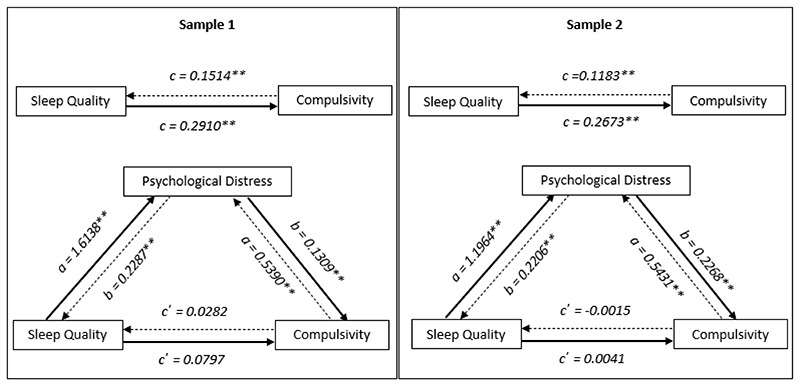
Sleep quality and compulsivity mediation models for Studies One (left) and Two (right). *significant results at p<0.05 level **significant results at p<0.01 level _______________ Indicates model where sleep quality predicts compulsivity, mediated by distress .............. Indicates model where compulsivity predicts sleep quality, mediated by distress

Figure 1 shows sleep quality as a predictor of compulsivity is mediated through psychological distress. Through mediation analyses on Sample One, we found the total effect of sleep quality on compulsivity to be significant (c path; *b* = 0.2910, t(744) = 5.8564, p<0.0001). The direct effect of sleep quality on compulsivity was not significant after accounting for distress (c’ path; *b* = 0.0797, t(744) = 1.2929, p = 0.1965) while the indirect effect of sleep on compulsivity was significant (a and b paths multiplied; *b* = 0.2113, 95% Confidence Interval (CI) = 0.1312 – 0.2961).

In Sample Two, we found the total effect of sleep quality on compulsivity to be significant (c path; *b* = 0.2673, t(791) = 5.0817, p<0.0001). The direct effect of sleep quality on compulsivity was not significant after accounting for distress (c’ path; *b* = -0.0041, t(791) = -0.0696, p = 0.9445), and the indirect effect of sleep on compulsivity was significant (a and b paths multiplied; b = 0.2713, 95% CI = 0.2031 – 0.3439). Taken together, our findings from Studies One and Two were consistent and indicate that that psychological distress is an important explanatory variable linking sleep quality and compulsivity.

When entering compulsivity as the predictor variable, we found these results to be bidirectional whereby psychological distress significantly mediated the relationship between compulsivity and sleep quality (Table 4).

### Diet quality and compulsivity

**Figure 2:**
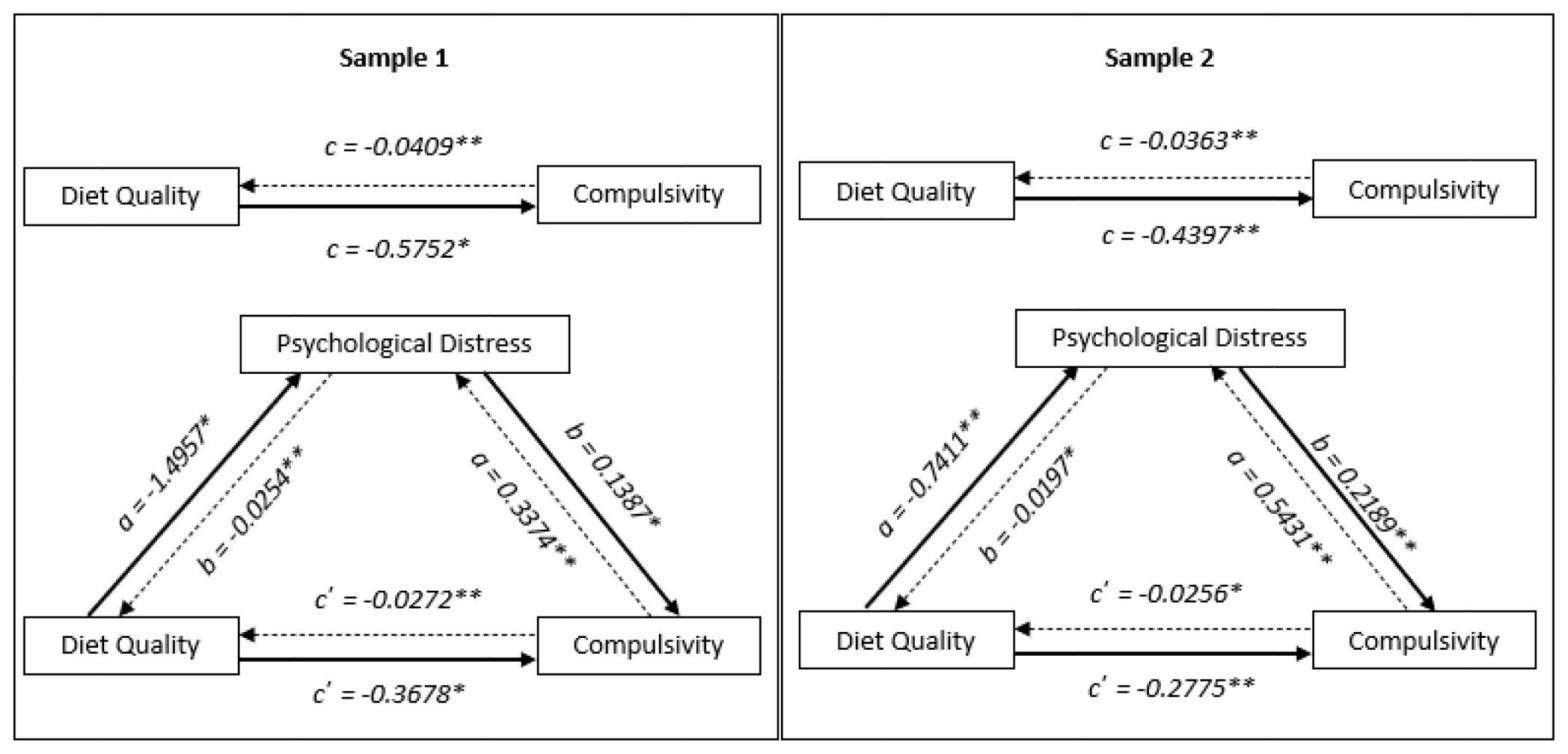
Diet quality and compulsivity mediation models for Studies One (left) and Two (right). *significant results at p<0.05 level **significant results at p<0.01 level _______________ Indicates model where diet quality predicts compulsivity, mediated by distress .............. Indicates model where compulsivity predicts diet quality, mediated by distress

Figure 2 shows diet quality as predictor of compulsivity is mediated by psychological distress. In Sample One, the total (c path; *b* = -0.5752, t(744) = -4.2317, p<0.0001), direct (c’ path; *b* = -0.3678, t(743) = -2.7383, p=0.0063) and indirect (a and b paths multiplied; *b* = -0.2074, 95% CI = -0.3162 - -0.1157) effects of diet quality on compulsivity were significant. These results indicate that psychological distress partially mediated the relationship between diet quality and compulsivity, while diet quality maintained some direct influence on compulsivity having accounted for distress.

Similarly in Sample Two, the total (c path; *b* = -0.4397, t(791) = -3.5821, p=0.0004), direct (c’ path; *b* = -0.2775, t(791) = -2.3788, p=0.0176) and indirect (a and b paths multiplied; *b* = -0.1622, 95% CI = -0.2611 - -0.0743) effects of diet quality on compulsivity were significant replicating our interpretation of the data in Sample One wherein psychological distress partially mediated the relationship between diet quality and compulsivity.

When entering compulsivity as the predictor variable, we again found the relationship between the lifestyle factor and compulsivity to be bidirectional. Specifically, compulsivity significantly predicted diet quality, with psychological distress partially mediating this relationship (Table 4).

### Physical activity and compulsivity

**Figure 3:**
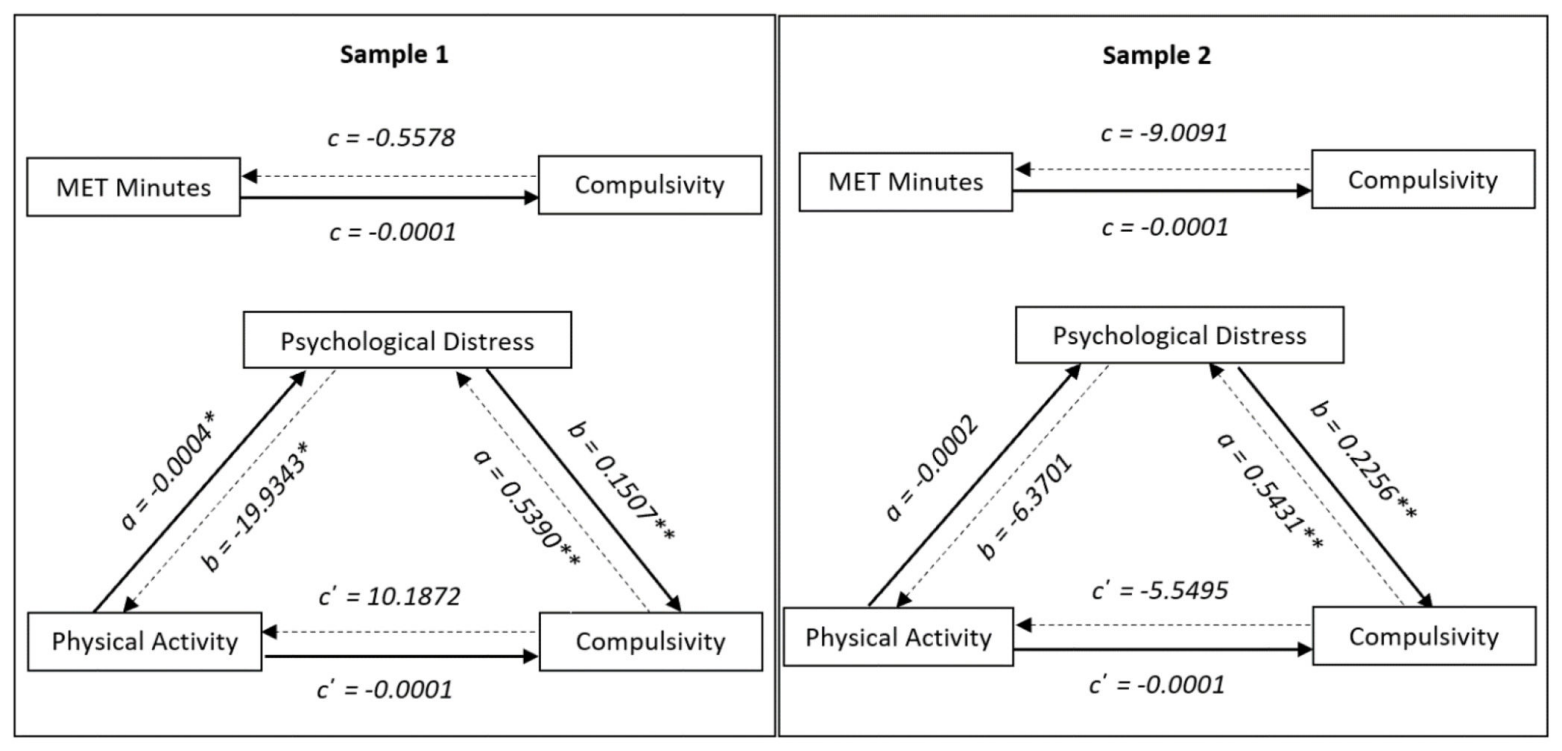
Physical activity and compulsivity mediation models for Studies One (left) and Two (right). *significant results at the p < 0.01 level **significant results at the p < 0.05 level _______________ Indicates model where physical activity predicts compulsivity, mediated by distress .............. Indicates model where compulsivity predicts physical activity, mediated by distress

Across Studies One and Two, we found no significant total effect of MET minutes on compulsivity. We also found no significant total effect of compulsivity on MET minutes. Please refer to Figure 3 for an illustration of the relationship between MET minutes and compulsivity as mediated by psychological distress.

## Discussion

In this study, we gathered data from two large independent samples and undertook mediation analyses to examine the relationship between lifestyle factors and compulsivity, and whether this relationship is statistically explained by psychological distress. In particular, we focussed our investigation on the lifestyle factors of sleep quality, diet quality and physical activity in separate mediation models. We found consistent results between the two samples. Our results showed an important role for psychological distress in explaining the bidirectional relationship between sleep quality and compulsivity, while psychological distress was somewhat important in explaining the bidirectional relationship between diet quality and compulsivity. Physical activity engagement did not predict compulsivity in either sample.

While some previous research has focused on the relationship between mental illness and lifestyle factors [56–58] including some compulsive disorders and lifestyle factors (for instance, OCD and its relationship with sleep [10, 59], dietary intake in gambling disorder [11], and exercise for the treatment of OCD [24]), this is the first analysis – to our knowledge - of the relationship between lifestyle factors and transdiagnostic compulsivity. Our results therefore have important implications for both the prevention of compulsive disorders in vulnerable populations, early intervention in compulsive behaviours and the potential treatment of compulsive disorders through the initial target of psychological distress, and secondarily through lifestyle interventions.

Our results suggest that psychological distress, and building the individual’s resilience to distress, are important targets for psychological therapy interventions for compulsive disorders. Given that psychological distress is linked to compulsivity, the reduction of psychological distress may help to mitigate compulsive tendencies and behaviours. Psychological distress can be targeted through a range of existing first-line therapies including acceptance and commitment therapy and cognitive-behavioural interventions, particularly exposure and response prevention in this clinical group. Psychological distress may also be targeted through trained and self-led strategies such as mindfulness meditation. The practice of mindfulness meditation has been shown to increase the individual’s resilience to psychological distress when undertaken as a short and intensive intervention [60, 61], as well as longer-term interventions with brief daily practice [62].

Sleep and diet are both closely related to psychological distress [34–36, 39], and diet has been directly linked to compulsivity. The differences in these relationships may be explained through differing pathways. While evidence suggests that diet may directly impact on BDNF levels [63] - a neurotrophic factor of which lower levels are associated with OCD [27, 28, 64], better sleep quality may improve resilience to psychological distress and emotion regulation which, in turn, may upregulate BDNF [32, 33]. Our findings support a theory that two direct paths to BDNF involve diet and psychological distress, while sleep is involved through an indirect pathophysiological pathway.

Given the role of sleep and diet in psychological distress and the direct relationship between diet and compulsivity, lifestyle interventions appear to be prime candidates for inclusion in stepped models of holistic psychiatric care. The more widespread adoption of sleep and/or diet interventions to clinical practice may be beneficial for people who do not respond to treatments targeting psychological distress. They may also be effective in bolstering gains made through interventions targeting psychological distress. When assessing symptom changes during the course of a therapy or intervention, we suggest that it is important to monitor changes in psychological distress.

We found that physical activity, measured in MET minutes, had no significant relationship with compulsivity in either sample. Currently, there is mixed evidence on the links between physical activity and compulsive symptoms and behaviours, with weak evidence for its benefits [21, 65]. Evidence from randomised controlled trials in OCD and substance use disorders suggests that planned and structured moderate- to high-intensity interval training may have the most profound impact on reducing compulsive behaviours and symptoms of compulsive disorders [24, 66]. However, MET minutes may be seen as a crude measure of physical activity as includes low-, moderate- and high-intensity activity without the total score distinguishing between these activities. Moreover, our measurement of *physical activity* - which includes the undertaking of incidental activities such as walking as a form of travel [67] - differs from existing research which suggests *physical exercise* - defined as planned and structured activity undertaken with the intention of improving physical fitness [67] - should be a specific target for reducing compulsivity.

Our findings hold transdiagnostic importance and are pertinent to both clinical populations and populations as-risk of developing compulsive disorders. It may be the case that early interventions for sub-clinical groups that target the reduction of psychological distress or the improvement of diet and/or sleep quality will be an effective preventative measure to lower an individual’s risk of developing a clinical compulsive disorder, especially in people with high transdiagnostic compulsive tendencies. As individuals recover from compulsive disorders, interventions targeting psychological distress and lifestyle factors may be an effective, acceptable and low-cost means of preventing relapse and maintaining physical health, with the latter being associated with significant morbidity and mortality in those experiencing with mental health problems [7].

The current study is not without limitations. We undertook analyses on cross-sectional samples and employed self-report measures. This limits our ability to assess causal pathways, and data may be impacted by the accuracy of participants’ reports of their own lifestyle, psychological distress and personality. We also conducted these studies in different countries during the COVID-19 pandemic. As health- socioeconomic- and cultural-related impacts of the pandemic were vastly different in Australia and the United States [68], differential effects are likely to have impacted the factors we studied across these groups, including lifestyle. While previous research has indicated a relationship between psychological distress and lifestyle factors [69, 70], we cannot necessarily generalise these results outside of Australia and the USA. Finally, we used different tools across studies to measure psychological distress and impulsivity. While we only used validated scales and our results suggest consistency across the two samples, it should be acknowledged that these tools may measure slightly different aspects of the same construct. For example, the S-UPPS-P measures the negative urgency aspect of impulsivity, while the BIS-11 does not. The K10 and DASS-21 also account for different psychological factors in the measure of psychological distress.

The current study has several strengths. We analysed data from two large independent samples, which provide evidence of the reproducibility of the results. The current study and findings we have presented are novel, and yet to be documented in the existing empirical literature. Specifically, we looked at three lifestyle factors comprehensively and maintained a transdiagnostic approach in order to provide broad clinical utility of the results relevant to the general population, as well as a range of compulsive disorders. We analysed samples across diverse socio-economic and cultural population and found consistent links between lifestyle and compulsivity. The consistency of results between the samples reflects the study’s methodological rigour. Indeed, an advantage of conducting this study across countries with different outcomes from the COVID-19 pandemic illustrates that the relationship between lifestyle and compulsivity may be pervasive across cultures. Mediation analyses allowed us to provide further insight into these relationships through the important mediating factor of psychological distress which may be an important target for intervention, along with lifestyle interventions.

## Conclusion

Looking ahead, there are a number of areas where research into the relationship between lifestyle factors and compulsivity/compulsive disorders may be required. Firstly, future research should investigate the aspects of diet which might be important to psychological distress and compulsivity such as adherence to a Mediterranean diet, or intake of particular non-inflammatory foods, for example [71]. Longitudinal studies assessing the aspects of lifestyle that lead to the transition to certain psychopathology will provide a clearer picture of the targets for patient education, early intervention and treatment for compulsive disorders. Future research may also focus more specifically on the measurement of high-intensity physical activity alone in order to focus the research towards understanding whether specific types of physical activity may reduce compulsive behaviours

## Supplementary Material

Supplementary material: tables

## Figures and Tables

**Table 1:** Constructs and corresponding instruments completed by each sample

	Sample One	Sample Two
**Compulsivity**		
Cambridge-Chicago Compulsivity Scale (CHI-T)	✓	✓
**Impulsivity**		
Short Form of the Barrett Impulsivity Scale-15 (BIS-11)	✓	
Short Urgency, Premeditation, Perseverance, Sensation Seeking, Positive Urgency, Impulsive Behavior Scale (S-UPPS-P)		✓
**Psychological distress**		
Depression, Anxiety and Stress Scale-21 (DASS-21)	✓	
K-10		✓
**Lifestyle factors**		
Pittsburgh Sleep Quality Index (PSQI)	✓	✓
Short Form Food Frequency Questionnaire (SFFFQ)	✓	✓
International Physical Activity Questionnaire (IPAQ)		
*Original version*	✓	
*Adapted version*		✓

**Table 2:** Participant characteristics

	Sample One (n=749)	Sample Two (n=796)
Age (mean, (SD))*	38.62 (12.54)	31.81 (12.25)
Sex (n (%))		
Female	395 (52.7)	422 (53.00)
Male	350 (46.7)	362 (45.50)
Other**	4 (0.5)	12 (1.60)
Employment (n)		
Full time*	427 (57.0)	232 (31.7)
Other (Part time, casual, student)*	241 (32.2)	452 (56.7)
Unemployed*	81 (10.8)	137 (17.2)
Clinical factors (mean, (SD))		
CHI-T*	24.07 (6.72)	26.75 (5.55)
DASS-21	13.10 (12.35)	
K10		22.00 (8.87)
BIS-11	32.95 (4.76)	
UPPS-P		42.71 (7.37)
DOCS	18.71 (14.88)	
OCI-R		6.13 (8.30)
Lifestyle factors (mean, (SD))		
PSQI	7.04 (4.57)	6.87 (3.64)
SF-FFQ*	9.94 (1.66)	11.29 (1.55)
IPAQ*	2353.35 (2444.03)	1616.81 (1845.87)

* indicates significant differences between groups at p<0.01

** indicates significant differences between groups at p<0.05

**Table 3:** Mediation results of the effect of lifestyle factors on compulsivity, mediated by psychological distress

	Total effect of lifestyle factor on compulsivity (*b*)	Total effect standard error	Total effect significance (p value)	Direct effect of lifestyle factor on compulsivity (*b*)	Direct effect standard error	Direct effect p value	Indirect effect of lifestyle factor on compulsivity (*b*)	Indirect effect standard error	95% confidence interval, lower	95% confidence interval, upper
**Sample One:**										
Sleep quality	0.2910	0.0297	**<0.0001**	0.0797	0.0617	0.1965	0.2113	0.0428	**0.1309**	**0.2961**
Diet quality	-0.5752	0.1359	**<0.0001**	-0.3678	0.1343	**0.0063**	-0.2074	0.0517	**-0.3179**	**-0.1128**
MET minutes	0.0000	0.0001	0.9687	0.0001	0.0001	0.4902	-0.0001	0.0000	-0.0001	0.0000
**Sample Two:**										
Sleep quality	0.2673	0.0526	**<0.0001**	-0.0041	0.0584	0.9445	0.2713	0.0355	**0.2031**	**0.3439**
Diet quality	-0.4397	0.1228	**0.0004**	-0.2775	0.1167	**0.0176**	-0.1622	0.0473	**-0.2611**	**-0.0743**
MET minutes	-0.0001	0.0001	0.4630	0.0000	0.0001	0.6720	0.0000	0.0000	-0.0001	0.0000

Predictor variable: lifestyle factors as stated in the table Outcome variables: compulsivity Mediator variable: psychological distress MET: metabolic equivalents of task

**Table 4:** Mediation results of the effect of compulsivity on lifestyle factors, mediated by psychological distress

	Total effect of lifestyle factor on compulsivity (*b*)	Total effect standard error	Total effect significance (p value)	Direct effect of lifestyle factor on compulsivity (*b*)	Direct effect standard error	Direct effect p value	Indirect effect of lifestyle factor on compulsivity (*b*)	Indirect effect standard error	95% confidence interval, lower	95% confidence interval, upper
**Sample One:**										
Sleep quality	0.1514	0.0259	**<0.0001**	0.0282	0.0218	0.1965	0.1233	0.0178	**0.0876**	**0.1588**
Diet quality	-0.0409	0.0097	**<0.0001**	-0.0272	0.0099	**0.0063**	-0.0137	0.0039	**-0.0218**	**-0.0068**
MET minutes	-0.5578	14.2021	0.9687	10.1872	14.7561	0.4902	-10.7450	4.6197	-20.5955	-2.1980
**Sample Two:**										
Sleep quality	.1183	.0233	**<0.0001**	-.0015	.0217	.9445	.1198	.0139	**.0939**	**.1476**
Diet quality	-.0363	.0101	**.0004**	-.0256	.0108	**.0176**	-.0107	.0040	**-.0192**	**-.0030**
MET minutes	-9.0091	12.2706	.4630	-5.5495	13.1042	.6720	-3.4596	4.9460	-12.9462	6.4851

Predictor variable: compulsivity Outcome variables: lifestyle factors as stated in the table Mediator variable: psychological distress MET: metabolic equivalents of task
