# Supplementary material for: The role of psychological distress in the relationship between lifestyle and compulsivity: An analysis of independent, bi-national samples
Source: CNS Spectr. Author manuscript; Available in PMC 2023 Sep 27. (PMC7614722; doi:10.1017/S1092852921001048)
Supplement: Supplementary material: tables [file EMS177423-supplement-Supplementary_material__tables.docx]

**Supplementary Table 1: Mediation results of the effect of lifestyle factors on obsessive-compulsive symptoms, mediated by psychological stress**

|  | Total effect of lifestyle factor on compulsivity (*b*) | Total effect  standard error | Total effect significance (p value) | Direct effect of lifestyle factor on compulsivity (*b*) | Direct effect standard error | Direct effect p value | Indirect effect of lifestyle factor on compulsivity (*b*) | Indirect effect standard error | 95% confidence interval, lower | 95% confidence interval, upper |
| --- | --- | --- | --- | --- | --- | --- | --- | --- | --- | --- |
| **Study 1:** | | | | | | | | | | |
| Sleep quality | 1.2445 | 0.1085 | **<0.0001** | 0.1814 | 0.1219 | 0.1371 | 1.0631 | 0.0982 | **0.8708** | **1.2577** |
| Diet quality | -1.2771 | 0.3152 | **0.0001** | -0.2391 | 0.2668 | .3703 | -1.0380 | 0.2110 | **-1.4635** | **-.6324** |
| MET minutes | .0003 | 0.0002 | 0.1605 | 0.0006 | 0.0002 | **0.0005** | -0.0003 | 0.0001 | -0.0006 | 0.000 |
| **Study 2:** | | | | | | | | | | |
| Sleep quality | .4838 | .0774 | **<.0001** | -.0705 | .0816 | .3880 | .5543 | .0725 | **.4226** | **.7041** |
| Diet quality | -.9434 | .1804 | **<0.0001** | <0.0001 | .1623 | **.0001** | -.3201 | .0907 | **-.5018** | **-.1469** |
| MET minutes | -.0001 | .0002 | .6480 | .0000 | .0001 | .9948 | -.0001 | .0001 | -.0002 | .0001 |
| Predictor variable: lifestyle factors as stated in the table  Outcome variables: obsessive-compulsive symptom severity  Mediator variable: psychological stress | | | | | | | | | | |

**Supplementary Table 2: Mediation results of the effect of obsessive-compulsive symptoms on lifestyle factors, mediated by psychological stress**

|  | Total effect of lifestyle factor on compulsivity (*b*) | Total effect  standard error | Total effect significance (p value) | Direct effect of lifestyle factor on compulsivity (*b*) | Direct effect standard error | Direct effect p value | Indirect effect of lifestyle factor on compulsivity (*b*) | Indirect effect standard error | 95% confidence interval, lower | 95% confidence interval, upper |
| --- | --- | --- | --- | --- | --- | --- | --- | --- | --- | --- |
| **Study 1:** | | | | | | | | | | |
| Sleep quality | 0.1207 | 0.0105 | **<0.0001** | 0.0164 | 0.0110 | 0.1371 | 0.1043 | 0.0088 | **0.0878** | **0.1220** |
| Diet quality | -0.0169 | 0.0042 | **0.0001** | -0.0045 | 0.0050 | 0.3709 | -0.0124 | 0.0031 | **-0.0189** | **-0.0064** |
| MET minutes | 8.6013 | 6.1226 | .1605 | 25.7870 | 7.4050 | **0.0005** | -17.6217 | -17.1857 | -25.9456 | -8.1828 |
| **Study 2:** | | | | | | | | | | |
| Sleep quality | .0973 | .0156 | **<0.0001** | -.0134 | .0155 | .3880 | .1107 | .0124 | **.0877** | **.1362** |
| Diet quality | -.0354 | .0068 | **<0.0001** | -.0294 | .0077 | **.0001** | .0060 | .0038 | -.0135 | .0013 |
| MET minutes | -3.7794 | 8.2756 | .6480 | -.0616 | 9.3670 | .9948 | -3.7179 | 4.7151 | -12.9581 | 5.6814 |
| Predictor variable: obsessive-compulsive symptom severity  Outcome variables: lifestyle factors as stated in the table  Mediator variable: psychological stress | | | | | | | | | | |
